# Socio-economic inequalities in malaria prevalence among under-five children in Ghana between 2016 and 2019: a decomposition analysis

**DOI:** 10.1186/s12936-025-05349-y

**Published:** 2025-05-08

**Authors:** Marian Yaa Abrafi Edusei, Olufunke Alaba, Denis Okova, Amarech Obse

**Affiliations:** 1 Health Economics Unit, School of Public Health, Faculty of Health and Sciences, University of Cape Town, Anzio Road, Observatory, 7925 South Africa; 2 German-West African Centre for Global Health and Pandemic Prevention, Kwame Nkrumah University of Science and Technology, Kumasi, Ghana; 3 The Institute for Life Course Development, University of Greenwich, London, UK

**Keywords:** Ghana, Malaria, Sub-Sahara Africa, Socio-economic inequalities, Under-five children

## Abstract

**Background:**

In a country with limited and unequally distributed resources, and plagued with malaria annually, under-five children are severely affected by this disease in Ghana. While the epidemiological burden of malaria on under-five children is well-documented, the extent and contributors of socio-economic inequalities in malaria prevalence remain under-explored. This study examined the intertemporal socioeconomic status (SES)-related inequalities in malaria prevalence among under-five children in Ghana from 2016 to 2019 and identified the key factors contributing to these disparities.

**Methods:**

Data were drawn from the 2016 and 2019 Ghana Malaria Indicator Surveys (GMIS). The study population consisted of under-five children who were tested for malaria in both surveys. Malaria prevalence served as the outcome variable, with the wealth index used as a proxy for socio-economic status. Socio-economic inequalities in malaria prevalence were evaluated using concentration indices and concentration curves. A decomposition analysis was employed to identify the socio-economic factors contributing to the observed inequalities.

**Results:**

A total of 2323 children in 2016 and 1938 children in 2019 were tested for malaria. Malaria prevalence increased from 8% in 2016 to 10% in 2019. The concentration index for 2019 (Concentration Index = − 0.224; Standard Error = 0.059; *p*-value = 0.000) was statistically significant and negative, indicating higher malaria prevalence among children from lower socio-economic backgrounds. However, the concentration index for 2016 (Concentration Index = − 0.052; Standard Error = 0.044; *p*-value = 0.230) was not statistically significant. In 2019, socio-economic status, region, and ethnicity accounted for 59.38%, 23.66%, and 4.46%, respectively, of the observed inequalities in malaria prevalence.

**Conclusion:**

The study revealed a persistent pro-poor inequality in malaria prevalence in under-five children in Ghana, underscoring the importance of targeted malaria control interventions. These interventions should prioritize socioeconomically disadvantaged groups to reduce inequalities in malaria prevalence which contributes to the 2030 Sustainable Development Goals of improving health (SDG 3) and reducing inequalities (SDG 10), among others.

**Supplementary Information:**

The online version contains supplementary material available at 10.1186/s12936-025-05349-y.

## Background

Malaria is a preventable and treatable infectious disease that remains a pressing public health problem significantly affecting under-five children and pregnant women worldwide [1–3]. In 2023, approximately 76% of all malaria cases occurred in under-five children [2–4], while 597,000 malaria related deaths were recorded in 83 endemic countries [2, 3]. Beyond mortality, malaria has a significant long-term impact, influencing individuals throughout their lives. It results in adverse pregnancy outcomes (e.g., low birth weight, maternal anaemia) and impedes children’s physical and cognitive development as well as impacts school attendance [5–8].

In Ghana, the impact of malaria is profound in children. Nearly 40% of all outpatient department (OPD) visits and approximately 47% of under-five fatalities in 2016 were due to malaria [9]. The disease accounted for approximately 11,000 deaths in 2018 [10]. While, malaria remains a leading cause of mortality and morbidity, among under-five children in Ghana, OPD cases of all malaria have fallen to 20% in 2022 [11], whilst its prevalence in under-five children has decreased from 14 to 9% between 2019 and 2022 [12]. This evidence indicates the progress towards malaria elimination in the country. However, the disparity in malaria prevalence across the country and socio-economic impact persist, affecting households [11]. For instance, malaria prevalence in 2022 ranged from 2% in the Greater Accra region to 15% in the Oti region, with rural areas reporting a threefold higher prevalence (12.8%) among under-five children compared to urban areas (4.3%) [13].

The economic burden of malaria on households in Ghana cannot be overstated, particularly among vulnerable populations [14]. In 2017, households spent US$ 20.29 per malaria episode for children under-five consisting of direct (US$9.54) and indirect costs due to lost income (US$11.10) [15]. Despite the implementation of Ghana’s National Health Insurance Scheme (NHIS), which covers over 95% of all common diseases [16], rural populations and lower-income households, some of whom live on less than US$ 1.90 per day as of 2023 [17], continue to face financial challenges when seeking malaria treatment, including indirect costs and lost income due to transportation and time spent accessing care respectively [18, 19].

Individuals with low socioeconomic backgrounds bear the brunt of the disease as confirmed by studies in sub-Saharan Africa (SSA) [22–22]. However, some studies found no significant association between malaria prevalence and socioeconomic status [23], while others suggest higher malaria rates among high-income populations [24, 25]. Nonetheless, low wealth status increased the odds of malaria infection (odds ratio [OR] 2.06, 95% CI 1.42–2.97, P < 0.001) in under-five children as shown by a systematic review of studies conducted in countries such as, Yemen, Burkina Faso, The Gambia, Ghana, Kenya, Uganda, Malawi, Equatorial Guinea, Ethiopia and Tanzania [21, 26], while mothers’ higher educational levels was found to be a protective factor against childhood malaria in Angola and Uganda [27].

While studies in socio-economic status (SES)-related inequalities in malaria prevalence among under-five children in Ghana are limited, studies in other countries in SSA including Nigeria [28], Ethiopia [29] and Kenya [30] found malaria prevalence to be concentrated in poorer populations (pro-poor inequalities) whereas malaria interventions utilization concentrated within the richer populations (pro-rich inequalities). Despite global efforts to reduce malaria incidence, socio-economic factors like wealth status, place of residence, distance to facilities, and maternal education in Anjorin et al*.* [31] study contributed to SES-related inequalities with 30.9% of poorer population having malaria in 11 SSA countries compared to 6.2% in the richest population [31]. This continues to influence prevalence rates in these vulnerable populations. Furthermore, understanding the temporal trends of inequalities provides essential insights to policymakers on whether inequalities are widening or narrowing [28, 32, 33]. Existing studies in Ghana have explored the socio-economic determinants of malaria prevalence in under-five children at specific time periods. This study provides insights into SES-related inequalities in malaria over time [9, 34, 35]. This study assessed the intertemporal socioeconomic inequalities in malaria prevalence between 2016 and 2019 and identifies the populations characteristics contributing to these disparities. The findings from the study can inform, Ghana's efforts at achieving the Sustainable Development Goals (SDGs) by reducing inequalities (SDG 10), combating infectious diseases, and decreasing neonatal and child mortality (SDG 3, targets 3.2 and 3.3) by 2030.

## Methods

### Data source

Data were sourced from the Ghana Malaria Indicator Survey (GMIS) of 2016 and 2019, focusing on under-five children [36, 37]. The GMIS is a nationally representative data that covers regional, urban, and rural populations. The main objectives of the GMIS are to determine the utilization and ownership of mosquito bed nets, estimate malaria prevalence and anaemia in pregnant women and children aged 6–59 months, and provide vital malaria indicators to assist in policies and strategies for malaria control in the country [37]. The survey employed the 2010 population and housing census as a sampling frame [38]. This study focused exclusively on the ten administrative regions (Ashanti, Central, Brong Ahafo, Western, Eastern, Upper East, Upper West, Volta, Greater Accra and Northern) of Ghana, although six new regions were created in 2019, as the geographic boundaries of the newly established regions were not defined at the time of the 2019 GMIS [38].

A total of 6000 households were sampled for 2016 and 2019 GMIS that were selected through a two-stage stratified sampling procedure [38]. At the first stage of the sampling process, regions were divided according to rural and urban locations yielding 20 strata. Next, enumeration areas (EAs), which identify the geographical households’ areas selected from the 20 strata. In the second stage, 30 households were sampled in each EAs [38]. The survey collects various information regarding malaria treatment, prevention, and prevalence and background information on characteristics of household and household members. Specifically, information such as age of child and mother, sex, maternal education, regions, place of residence, household wealth, and the relationship of household members to the head of the household. Moreover, information on dwelling characteristics including building materials, toilet facilities, roofing, ownership, and coverage of insecticide-treated nets (ITNs), intermittent preventive treatment (IPT), indoor residual spraying and knowledge of malaria were collected using the household questionnaire, women’s questionnaire, and biomarker questionnaire [36]. All datasets were available publicly from the Demographic and Health Survey (DHS) programme.

### Study population

While the GMIS sampled 6000 households, the number of under-five children (6–59 months) included in the surveys was 2323 (2016) and 1938 (2019).

### Study variables

#### Outcome variable

The outcome variable for this study was the malaria test result. As part of the GMIS, malaria tests are done using either rapid diagnostic tests (RDT) or microscopy. For microscopy, which is still considered the gold standard by the Centre for Disease Control and Prevention (CDC) [39], thick blood smear samples are taken and diagnosed in a laboratory by microscopists for the presence of *Plasmodium* parasites. Due to limited trained microscopists in the field, RDT is also conducted with blood samples taken and diagnosed by a standard packaged sample applicator [37, 40] on the field.

The outcome variable was a binary variable indicating whether a child tested positive (coded = 1) or negative (coded = 0) for malaria. However, malaria test results were not reported for 91.93% and 90.42% of the children tested for malaria during GMIS in 2016 and 2019, respectively. Therefore, two separate analyses of inequality were conducted: (a) among children with reported malaria test plus proxy malaria test result for children without reported malaria test, (b) among children with reported malaria test result. The proxy malaria test result in the first analysis was computed based on fever occurrence among the children with missing test result. If malaria blood test results were not reported and the child tested negative for fever, the proxy malaria test results were set to negative (0) because the absence of fever indicates no active malaria infection in the child [35, 41]. If the child tested positive for fever (1) and malaria blood test results were not reported, the observation was excluded because the presence of fever without a malaria diagnosis could mean other conditions (e.g. anaemia) [35, 41].

#### Explanatory variables

The Commission of Social Determinants of Health (CSDH) framework by Solar and Irwin [42] was applied the study. Empirical review of related literature [28, 35, 41, 45–47] informed the choice of explanatory variables that contribute to inequality in malaria prevalence. The CSDH framework comprises three main components, the structural and social determinants of health inequities, intermediary determinants and social determinants of health and impact on equity in health and well-being. These components consist of socioeconomic position, social class, education, occupation, income, and material circumstances that affect an individual’s well-being. The selected explanatory variables include characteristics of a child, mother and households, including wealth, place of residence, mother’s age, ethnicity, child’s age, mother’s education, and national health insurance coverage for the child. Table 1 describes the variables in detail.

**Table 1:** Description of study variables

Variables	codes	Description
Outcome Variable		
Malaria prevalence	0 = negative, 1 = positive	Malaria test result
Explanatory Variable		
Wealth quintile	0 = poorest 1 = poorer 2 = middle 3 = richer 4 = richest	Household socioeconomic status measured by wealth
Ethnicity	0 = Akan 1 = G/Dangme 2 = Ewe 3 = Guan 4 = Mole-Dagbani 5 = Grusi 6 = Gurma 7 = Mande	Ethnicity of respondents
Age of child	0 = 0–12 months 1 = 13–24 months 2 = 25–36 months 3 = 36–48 months 4 = 49–59 months	Age of the child in months
National Health Insurance Scheme (NHIS) membership of child	0 = No 1 = Yes	Current health insurance coverage
Under-five children sleeping in treated net	0 = No 1 = Yes	Whether a child slept under an insecticide net the previous night
Ownership of household net	0 = No 1 = Yes	Whether households have any mosquito nets
Maternal education	0 = No formal education 1 = Primary 2 = Secondary 3 = Higher	Mother’s highest level of education
Mother’s age	0 = 15–24 years 1 = 25–34 years 2 = 35–44 years 3 = 45–49 years	Mother’s age in years
Place of residence	0 = Rural 1 = Urban	Current place of residence
Region	0 = Western 1 = Central 2 = Greater Accra 3 = Volta 4 = Eastern 5 = Ashanti 6 = Brong Ahafo 7 = Northern 8 = Upper East 9 = Upper West	Regional location

#### Living standard indicator

The GMIS uses a principal component analysis (PCA) to generate household wealth index which is an indicator of living standard or socioeconomic status [33]. The wealth index is computed based on household assets and possessions ranging from televisions, bicycles, automobiles, and housing characteristics like ceilings and floor types [37]. The wealth index from the GMIS was used for ranking the study population according to their wealth status from the poorest to the richest.

### Data analysis

All analysis and data management were conducted using the Stata software version 15. All samples from 2016 and 2019 were pooled to test for chi-square test analysis. Descriptive statistics were computed using the chi-square analysis to test the differences between the variables associated with malaria prevalence between 2016 and 2019 [48]. For each year, concentration index computation and decomposition analysis were conducted separately. The study accounted for the complex sampling procedure by using (svyset) in Stata command. Values with *p*-value less than 0.05 were considered significant [48].

### Measuring socioeconomic status (SES)-related inequality in malaria prevalence

Concentration curves (CC) and concentration indices (CI) were used to analyse socioeconomic status (SES)-related inequalities in malaria prevalence in under-five children [49, 50]. While inequalities can be assessed using statistical tests or regression analyses, such analyses only provide estimates of group differences. Inequality analysis using CI (and CC) considers the experiences of the entire population rather groups, furthermore, CI provides quantitative measure of the inequality which can be compared between different time periods [33]. The CC plots the cumulative proportion of malaria prevalence (y-axis) against the cumulative proportion of under-five children, ranked by their socio-economic status from the poorest to the richest (x-axis) [50]. If there is no inequality, the CC lies on the 45-degree diagonal line that runs from the origin to the top right-hand corner, known as the line of equality. Pro-poor inequality exists when the CC lies above the line of equality, indicating that malaria is disproportionately prevalent among the poorest, and vice versa [49]. While the CC provides a visual display of the inequality in malaria prevalence, the CI was used to quantify the degree of the inequality. The magnitude of the CI for a quantitative variable falls within the boundaries of − 1 and + 1, with a negative and positive value indicating a disproportionate concentration of malaria in the poorest (pro-poor inequality) or richest (pro-rich inequality) population, respectively. A value of zero indicates no inequality or an indeterminate case where the concentration curve crosses the line of equality [33]. However, for a binary outcome variable, the boundaries of the CI are (*μ* −1) and (1- *μ),* where μ is the mean of the outcome variable. Wagstaff [49] and Erreygers [51] proposed normalization of the CI for binary health outcomes so that the value falls within the − 1 and + 1 limits. However, recently, it has been shown that such normalization may give counterintuitive results for policy interpretation [52]. Therefore, the standard concentration index (non-normalized) was used for this study.

The mathematical expression of the CI using the convenient covariance is given as [33]

1 $${\text{CI}} = \frac{2}{\mu } {\text{cov}} \left( {H,R} \right),$$

where CI is the concentration index; $H,$ is the health variable; $R$ is the fractional rank of individuals in the living standards distribution; *μ* is the mean of the health variable; and *cov* $\left( {H,R} \right)$ is the covariance between the health variable and the fractional rank of living standards of individuals.

### Decomposition of the concentration index

The concentration index, CI*,* of malaria prevalence was decomposed into the multiple contributing factors [33], to explain factors contributing to the malaria prevalence inequalities in under-five children. The contribution of each explanatory variable (e.g., sex, age) to malaria prevalence inequality, was obtained as a product of the sensitivity (elasticity) of malaria prevalence to the changes in explanatory variable and concentration index of the explanatory variable, the latter representing socioeconomic inequality in the distribution of the explanatory variable.

A linear additive regression model of malaria prevalence, $y$, consisting of a set of K determinants, $X_{K}$, can be expressed as [33]

2 $$y = a + \sum_{K} \beta_{K} X_{K} + \varepsilon ,$$

Given the regression model in Eq. 2, the concentration index $CI$ can be redefined as:

3 $$CI_{ } = \mathop \sum \limits_{k} \left( {\frac{{\beta_{k} \overline{x}_{k} }}{\mu }} \right)CI_{k} + \frac{{GCI_{\varepsilon } }}{\mu },$$

where $\mu$ is the mean of malaria prevalence (y); $\overline{x}_{k}$ is the mean for the explanatory variable, $x_{k}$; $\left( {\frac{{\beta_{k} \underline {x}_{k} }}{{\upmu }}} \right)$ is the elasticity of malaria prevalence with respect to changes in the determinant variable $x_{k}$; and $CI_{k}$ is the concentration index of $x_{k}$ variable. The product of $\left( {\frac{{\beta_{k} \underline {x}_{k} }}{\mu }} \right)$ and $CI_{k}$ gives contribution of each determinant $x_{k}$ to overall inequality. $GCI_{\varepsilon }$ denotes the generalized concentration index for the residual (ε). The error term (residual) captures wealth-related inequality not accounted for by systematic variations in the determinants across the study population [33]. For a well-defined model, it is expected that the residual term approaches zero [33].

Bootstrapping with 1000 replications was employed to estimate standard errors. Furthermore, the standard errors were adjusted for sampling weights and the multi-stage sampling design procedures ensure statistical accuracy.

## Results

### Socio-demographic characteristics of the study population

A total of 2323 and 1938 under-five children, were tested for malaria during the 2016 and 2019 GMIS, respectively. The prevalence of malaria among children increased by 2% points over the 3 years, from 8 to 10%. Greater proportions of the children included in the study were under the age of 12 months, both in 2016 (23.68%) and in 2019 (25.64%). Mothers’ education status improved between the study years, with a 7%-point reduction in the fraction of children that have mothers with no formal education. In terms of ethnicity, 34.44% (2016) and 36.89% (2019) of the study participants belonged to Akan and Mole-Dagbani ethnic groups. Furthermore, above 60% of the participants resided in the rural areas in both years. The Northern region accounted for the highest share of the study participants, which decreased slightly over the years, from 17.39% to 15.22%. The lowest share of participants, 6.80% (2016) and 8.62% (2019), were from the Central region (Table 2). There was no statistically significant difference in the socio-demographic characteristics of the study population of 2016 and 2019 except for ethnicity, maternal education and NHIS coverage of child.

**Table 2:** Socio-demographic characteristics of study sample between 2016 (N = 2323) and 2019 (N = 1938)

Variables characteristics	Headcount 2016 n (%)	Headcount 2019 n (%)	Chi-square statistic of difference
**Socioeconomic status (wealth quintile)**			0.932
Poorest	480 (20.66)	371 (19.14)	
Poorer	478 (20.58)	373 (19.25)	
Middle	472 (20.32)	393 (20.28)	
Richer	438 (18.85)	388 (20.02)	
Richest	455 (19.59)	413 (21.31)	
**Ethnicity**			0.020**
Akan	800 (34.44)	656 (33.85)	
Ga/Dangme	105 (4.52)	87 (4.49)	
Ewe	272 (11.71)	241 (12.44)	
Guan	89 (3.83)	49 (2.53)	
Mole Dagbani	647 (27.85)	715 (36.89)	
Grusi	119 (5.12)	86 (4.44)	
Gurma	249 (10.72)	85 (4.39)	
Mande	42 (1.81)	19 (0.98)	
**Maternal education**			0.083*
No formal education	792 (34.00)	515 (26.57)	
Primary	465 (20.02)	393 (20.28)	
Secondary	926 (39.86)	923 (47.63)	
Higher	140 (6.03)	107 (5.52)	
**Place of residence**			0.883
Rural	1453 (62.55)	1172 (60.47)	
Urban	870 (37.45)	766 (39.53)	
**National health insurance scheme (NHIS) coverage of child**			0.034**
Yes	1525 (65.65)	1220 (62.95)	
No	798 (34.35)	718 (37.05)	
**Regions**			0.821
Western	172 (7.40)	182 (9.39)	
Ashanti	234 (10.07)	189 (9.75)	
Eastern	184 (7.92)	153 (7.89)	
Central	158 (6.80)	167 (8.62)	
Brong Ahafo	215 (9.26)	157 (8.10)	
Northern	404 (17.39)	295 (15.22)	
Volta	257 (11.06)	170 (8.77)	
Greater Accra	206 (8.87)	167 (8.62)	
Upper East	278 (11.97)	227 (11.71)	
Upper West	215 (9.26)	231 (11.92)	
**Ownership of household insecticide net**			0.319
No	267 (11.49)	225 (11.61)	
Yes	2056 (88.51)	1713 (88.39)	
**Under-five slept under insecticide net previous night**			0.245
Yes	1494 (64.31)	1299 (67.03)	
No	829 (35.69)	639 (32.97)	
**Mother’s age**			0.977
15–24 years	485 (20.88)	421 (21.72)	
25–34 years	1159 (49.89)	975 (50.31)	
35–44 years	626 (26.95)	503 (25.95)	
45–49 years	53 (2.28)	39 (2.01)	
**Child’s age**			0.399
0–12 months	550 (23.68)	497 (25.64)	
13–24 months	457 (19.67)	401 (20.69)	
25–36 months	482 (20.75)	374 (19.30)	
36–48 months	424 (18.25)	341 (17.60)	
49–59 months	410 (17.65)	325 (16.77)	
**Malaria prevalence**			0.144
Positive	208 (8.95)	204 (10.53)	
Negative	2115 (91.05)	1734 (89.47)	

**, and * indicate the statistical significance at 95%, and 90% confidence intervals

### Socioeconomic status-related inequality in malaria

**Fig. 1:**
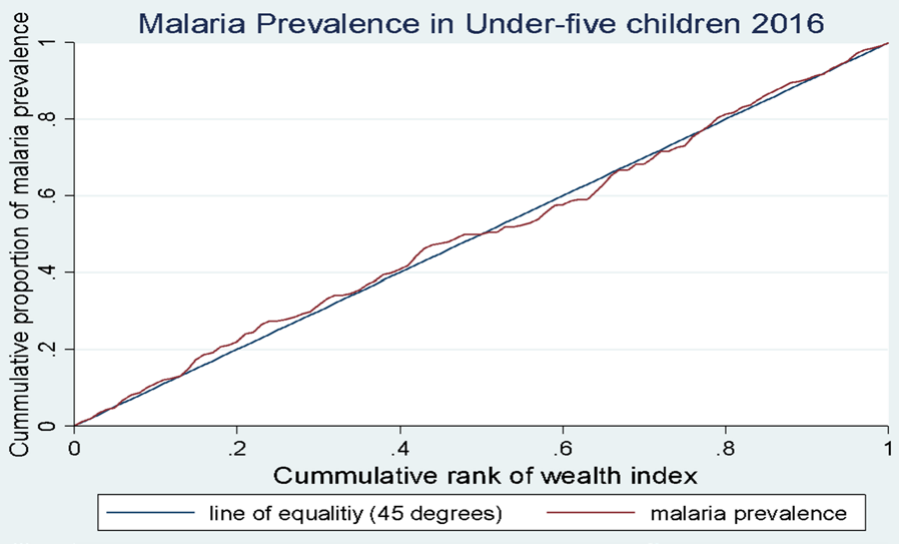
Concentration curve for prevalence in malaria in under-five children in 2016

**Fig. 2:**
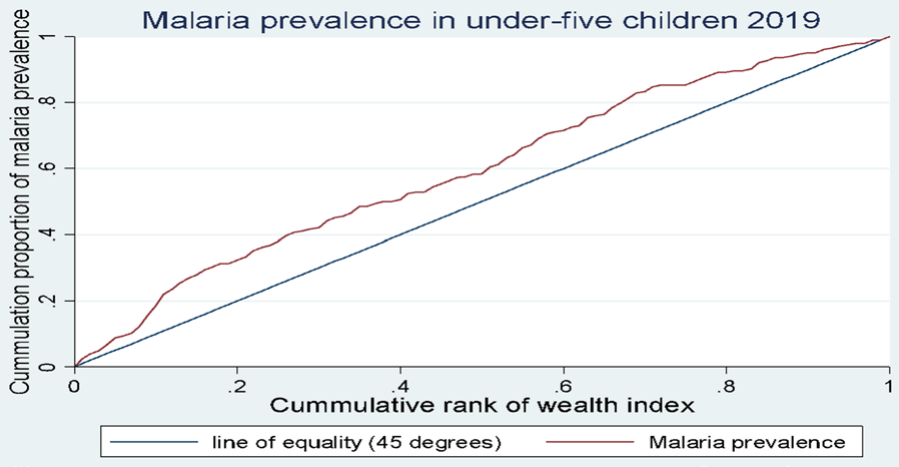
Concentration curve for prevalence in malaria in under-five children in 2019

Table 3 presents concentration indices of malaria prevalence in the children with reported or proxy malaria results. The concentration index of malaria prevalence in 2016 was found to be statistically insignificant (CI = − 0.052, SE = 0.053, *p*-value = 0.230) indicating no evidence of inequality which is supported by the concentration curve that crossed the line of equality (Fig. 1). The concentration curve of the 2019 malaria prevalence lies above the line of equality, suggesting pro-poor inequality in malaria prevalence (Fig. 2). This was corroborated by a statistically significant concentration index, (CI = − 0.224, SE = 0.590, *p*-value = 0.000), which confirmed the disproportionate prevalence of malaria among Ghanaian under-five children with low socioeconomic status in 2019.

**Table 3:** Concentration indices for prevalence of malaria in under-five children in Ghana for 2016 and 2019 in children with reported and proxy malaria result

Year	Observations	Concentration index	Standard error	*p*-value
2016	2323	− 0.052	0.053	0.230
2019	1938	− 0.224	0.590***	0.000

***significant at 99% confidence interval based on bootstrap standard errors with 1000 replications

Table 4 presents malaria test results among children with reported malaria test. The concentration index in 2016 was found to be statistically insignificant (CI = − 0.009, SE = 0.017, *p*-value = 0.591). The 2019 concentration index was statistically significant (CI = − 0.046, SE = 0.019, *p*-value = 0.017) indicating evidence of inequality in the poorer population.

**Table 4:** Concentration indices for prevalence of malaria in under-five children in Ghana for 2016 and 2019 among children with reported malaria result

Year	Observations	Concentration Index	Standard Error	*p*-value
2016	252	− 0.009	0.017	0.591
2019	256	− 0.046	0.019**	0.017

**significant at 95% confidence interval based on bootstrap standard errors with 1000 replications

### Decomposition of concentration index

Table 5 illustrates the result of the decomposition of the concentration index of malaria prevalence in 2019 among children with either reported or proxy malaria prevalence. The table depicts elasticities, concentration indices, and contributions of the socioeconomic and demographic factors included in the decomposition analysis. A total sample size of 1938 under-five children in 2019 was decomposed. Overall, socioeconomic status (wealth), region, and ethnicity significantly contributed to the inequality in 2019. Household wealth explained greater part (59.38%) of the inequality. The second largest contributor to the inequality was residential region, which explained 23.66% of the inequality. Mother’s education was the third key factor explaining 7.14% of the disparity in prevalence of malaria. Living in urban areas contributed by reducing inequality by 4.02%. Ownership of insecticide treated household nets and NHIS coverage of child, contributed, 0.89% and 1.78%, respectively. The unexplained factors captured by the residual accounted for 7.59% of the inequality although statistically insignificant. The included socio-demographic variables explained 92.41% of the observed inequality in malaria prevalence at 99% confidence level. The concentration index of malaria prevalence for 2016 was not decomposed because it was found to be statistically insignificant. Adjusted standard errors for the decomposition of the concentration index of malaria prevalence could not be obtained for observations with reported malaria test results (excluding proxy test results) in 2016 and 2019 due to small sample sizes. However, the unadjusted results for 2019 shows that, urban residence and wealth status contributes to 80.43% and 32.61% of the overall inequality. Region explained 26.08% of the inequality. The residual not statistically significant, explained 56.52% of the unexplained factors. The 2016 concentration index was statistically not significant hence, no decomposition.

**Table 5:** Decomposition of Concentration Index for Malaria Prevalence for Under-Five Children in Ghana, 2019

Explanatory variables	Elasticities (SE)	Concentration indices (SE)	Contributions (SE)	Total contributions	Percentage of total contribution
**Wealth quintile**					
**Poorest (Reference)**					
Poorer	− 0.098 (0.058) *	− 0.200 (0.023) ***	0.019 (0.012)		
Middle	− 0.065 (0.045)	0.217 (0.021) ***	− 0.014 (0.009)		
Richer	− 0.075 (0.041) *	0.549 (0.017) ***	− 0.041 (0.022) *	− 0.133	59.38%
Richest	− 0.114 (0.041) ***	0.852 (0.008) ***	− 0.097 (0.035) ***		
**Household Net Ownership**					
**No (Reference)**					
Yes	0.129 (0.194)	− 0.014 (0.005) ***	− 0.002 (0.003)	− 0.002	0.89%
**Under-Five Net Use**					
**No (Reference)**					
Yes	− 0.043 (0.126)	− 0.094 (0.009) ***	0.004 (0.012)	0.004	− 1.78%
**NHIS Coverage of child**					
**No (Reference)**					
Yes	− 0.084 (0.099)	0.041 (0.011) ***	− 0.004 (0.004)	− 0.004	1.78%
**Ethnicity**					
**Akan (Reference)**					
Ga/Dangme	− 0.002 (0.009)	0.485 (0.057) ***	− 0.001 (0.004)		
Ewe	− 0.018 (0.021)	0.346 (0.033) ***	− 0.006 (0.007)		
Guan	− 0.023 (0.009) **	0.078 (0.061)	− 0.002 (0.002)		
Mole Dagbani	0.001 (0.133)	− 0.219 (0.013) ***	− 0.000 (0.029)	− 0.01	4.46%
Grusi	− 0.039 (0.017) **	− 0.185 (0.055) ***	0.007 (0.004) *		
Gurma	0.034 (0.021)	− 0.194 (0.055) ***	− 0.007 (0.005)		
Mande	− 0.012 (0.004) ***	0.066 (0.179)	− 0.001 (0.002)		
**Residence**					
**Rural (Reference)**					
Urban	− 0.038 (0.113)	− 0.243 (0.011) ***	0.009 (0.028)	0.009	− 4.02%
**Region**					
**Western (Reference)**					
Central	− 0.013 (0.008)	0.346 (0.034) ***	− 0.005 (0.003)		
Greater Accra	− 0.041 (0.013) ***	0.782 (0.017) ***	− 0.032 (0.010) ***		
Volta	− 0.030 (0.025)	0.171 (0.038) ***	− 0.005 (0.004)		
Eastern	− 0.059 (0.023) **	0.393 (0.034) ***	− 0.023 (0.009) **		
Ashanti	− 0.095 (0.033) ***	0.552 (0.026) ***	− 0.053 (0.018) ***		
Brong Ahafo	− 0.077 (0.038) **	0.148 (0.033) ***	− 0.011 (0.006) *	− 0.053	23.66%
Northern	− 0.189 (0.080) **	− 0.097 (0.026) ***	0.018 (0.009) *		
Upper East	− 0.008 (0.075)	− 0.385 (0.032) ***	0.003 (0.028)		
Upper West	− 0.185 (0.083) **	− 0.295 (0.028) ***	0.055 (0.025) **		
**Maternal Education**					
**No Formal education (Reference)**					
Primary education	0.018 (0.049)	− 0.099 (0.028) ***	− 0.002 (0.005)		
Secondary education	− 0.093 (0.077)	0.218 (0.018) ***	− 0.020 (0.017)	− 0.016	7.14%
Higher education	0.008 (0.015)	0.739 (0.032) ***	0.006 (0.011)		
**Age of Mother**					
**15–24 years (Reference)**					
25–34 years	− 0.048 (0.087)	0.068 (0.015) ***	− 0.003 (0.006)		
35–44 years	− 0.028 (0.055)	− 0.033 (0.026)	0.001 (0.002)	− 0.006	2.68%
45–49 years	0.019 (0.015)	− 0.226 (0.091) **	− 0.004 (0.005)		
**Age of Child**					
**0–12 months (Reference)**					
13–24 months	0.163 (0.040) ***	0.003 (0.028)	0.000 (0.004)		
25–36 months	0.156 (0.039) ***	0.037 (0.030)	0.006 (0.005)		
36–48 months	0.064 (0.034) *	− 0.025 (0.031)	− 0.001 (0.002)	0.004	− 1.78%
49–59 months	0.051 (0.031)	− 0.035 (0.033)	− 0.001 (0.002)		
**Explained Contribution**				− 0.207***	92.41%
**Residual**			− 0.017 (0.014)	− 0.017	7.59%
**Total**				− 0.224	100%

*Etimated sample size was 1938; standard errors in parenthesis boostraped using 1000 replications adjusting for sampling design*

***, **, * indicate the statistical significance at 99%, 95%, and 90% confidence intervals

## Discussion

There is substantial evidence on SES-related health inequalities in low- and middle-income countries (LMICs) [53] although the evidence is sparse in some contexts, including prevalence of malaria in under-five children [35]. Still, continued evidence is needed to understand the dynamics of inequality including changes over time and contributors to the inequalities to inform policies and assess progress to SDGs. This study demonstrated that malaria was disproportionately prevalent among under-five children with poorer socioeconomic status in 2019 in Ghana while there was no evidence of inequality in 2016 based on the GMIS data. The 2019 result agrees with findings from a multi-country study from sub-Saharan Africa (SSA) [54] which showed concentration of malaria in socioeconomically disadvantaged under-five children between 2003 and 2016. While the burden of malaria had reduced overtime, the poorest remain with highest risk of infection in endemic countries [21, 34, 55, 56] due to poor knowledge of the use of the preventive interventions (e.g. insecticides nets), housing units and locations that are characterized by slums, which are breeding sites for mosquitoes. A study in Madagascar found inequalities in malaria prevalence concentrated amongst the poorest population as a result of differences in the knowledge of the disease [57]. Although most studies in the SSA region found the poorer households being mostly affected by malaria, a study from Nigeria proved otherwise [24]. The study indicated malaria prevalence being concentrated in the better offs than poorer households. This study used self-reported malaria cases [24] which might not give an accurate measure of malaria prevalence due of recall bias and differences in knowledge of symptoms of malaria among the population.

About 90% of the inequality in the prevalence of malaria among the Ghanaian under-five children in 2019 in this study was explained by household wealth (59.38%), regional residence (23.66%), and maternal education (7.14%). The lower likelihood of malaria prevalence in under-five children from richer households compared to the poorer was reported by other studies from Ghana [5, 9, 20]. The largest and significant contribution of wealth status to SES-related inequality in health has been well established [55, 58]. Accordingly, other studies found wealth status as the largest contributor to inequality and differences in inequality between groups in under-five malaria prevalence [27, 29, 59]. For instance, Edwin et al. [59] in Nigeria found household wealth explaining 68% of under-five malaria infection using maternal education as a measure of socioeconomic status while 6% was attributed to regional differences, using maternal education as a proxy for socioeconomic status [59]. Another study assessing differences in inequality in under-five malaria between children with educated and non-educated mothers, found wealth status to be the largest contributor of the difference albeit with lower magnitude (26%) in Angola, Tanzania, and Uganda [27]. The contribution of wealth status for such inequalities has been attributed to richer households being able to afford basic healthcare needs and provision of conducive environments such as quality housing, indoor residual spraying, and protective clothing for their children, which contributes to better health outcomes [60].

Regional residence was the second most important variable contributing to inequality in malaria prevalence in children. Regional differences as risk of malaria have been previously reported for Ghana [6, 34, 56, 61], which has been explained by differences in climatic conditions that are favourable for mosquito breeding including temperature, humidity and availability of standing water bodies [56]. A spatial analysis of climatic influence on malaria prevalence in Ghana showed that regions that were experiencing high rainfall and high humidity had a positive association with malaria prevalence and incidence [34, 56]. Higher odds of malaria cases were reported for Ashanti, Central, Volta, Upper East, Upper West regions of Ghana than the Greater Accra and Western regions [6]. The Southern region of Ghana is less susceptible to droughts and floods [13]. Furthermore, it is more developed than the Northern regions with better roads, economic activities, and health and educational infrastructure [13]. A factor that is closely related to residential region is ethnicity and thus the contribution of, ethnicity may partly reflect the contribution of region [62]. The Northern region of Ghana is mainly characterized by the Grusis and Mole Dagbanis ethnic groups whilst, the Southern regions are characterized by the Akans and Ga/Dangmes [13]. A study from Bangladesh also found that vector distribution and the prevalence of malaria increased in ethnic tribes that stayed in places characterized by extensive rainfall and forest density compared to populations with less forest density [63]. Regarding residential areas, urbanicity of residence is also important in malaria inequality. This study found that living in rural areas increased inequality in malaria prevalence to the disadvantage of the poorer. This is because compared to urban settlements, rural locations, which are populated by the poor, are characterized by unfavourable environments that breed mosquitos [9, 64, 65]. For instance, Afoakwah *et.al* [9] found that vector transmission and malaria prevalence in under-five children in urban cities of Ghana are twice as low as in rural areas [9]. A similar study in Tanzania indicated a higher number of malaria cases in rural areas compared to urban locations [66]. Differences in housing materials between rural and urban areas could be among the potential factors influencing socioeconomic inequalities in malaria prevalence whereby urban residents, with higher socioeconomic status are likely to afford building materials that inhibit mosquito breeding and promote vector control [60]. Tusting et al*.* [60] indicated higher odds of malaria parasitaemia in houses with thatch and mud walls, which are mostly characterized in rural settings compared to most houses in urban areas with screened windows, cements, and fitted ceilings [60].

Maternal education was the third highest contributor having an increasing effect to the observed inequalities in malaria prevalence in children in 2019 in this study. The existing literature has already established the significant impact of a mother’s education to morbidity and mortality in children [59, 67]. Mother’s educational background is also significantly associated with childhood malaria infection in endemic regions [27, 59, 67]. The odds of malaria in children whose mothers have had a minimum of 6 years of schooling were reported to be low in the study covering nine SSA countries [67]. Afoakwah et al. [9] and Sarkodie et al.[68] also found lower rates of malaria infection in Ghanaian children whose mothers had at least a secondary education [9, 68]. Education is, therefore, an important lever to support the quest and ongoing activities in controlling malaria and eliminating it.

The main contributing factors to inequality in the prevalence of malaria in under-five children in this study can be well understood with the Social Determinants of Health (SDH) framework [42]. The SDH explains that the health of the population is affected by a wide range of personal (age, sex), environmental (e.g. neighbourhoods), and social factors (e.g. income, education) [42]. These factors are interconnected such that they result to social stratifications or socioeconomic hierarchies influenced by access to resources, power and prestige which then directly affect an individual’s health outcomes [42]. While medical services and vector control programmes are important to eradicating malaria, reducing inequalities in malaria prevalence needs approaches that address differences in the social determinants of health.

### Policy implications

This paper’s findings contribute to the growing body of literature on malaria by offering relevant policy insights as to the nature and magnitude of socioeconomic inequalities in malaria prevalence in under-five children in Ghana. It also supports two of the sustainable development goals of promoting health and well-being, SDG 3 and reduction of all forms of inequalities, SDG 10. Findings from the study provides a crucial support to the current Ghana National Malaria Elimination Strategic Plan’s (GNMESP) objectives by reducing socio-economic disparities in malaria prevalence through targeted and equitable distribution malaria interventions to populations in need.

### Strengths and limitations

The study employed the recently available datasets of the GMIS, which gives a good representation of the present information of malaria indicators and evidence. These datasets are also context specific to Ghana providing a nationally representative results and specific recommendations for policies. It is also one of the few papers to examine the socioeconomic inequalities in malaria prevalence using the concentration index and decomposition approach to identify factors contributing to the disparity in malaria prevalence.

Conversely, the study was not without limitations. First, both datasets from 2016 and 2019 had inadequate information on the results of malaria parasitaemia by microscopy testing. Therefore, a proxy outcome variable was generated from fever occurrence in the past 2 weeks and the results of malaria blood tests. Children without fever and no reported malaria test result was classified as children with no malaria. While fever is generally a key indicator of malaria, asymptomatic malaria can also be present without fever [35, 69, 70]. Therefore, considering children without fever as not having malaria may have underestimated prevalence of malaria in the analysis. It is not clear how this may have affected the inequality of malaria prevalence. However, an analysis of inequality has also been presented exclusively among children with reported malaria test results. The results are comparable with insignification inequality for 2016 and pro-poor inequality in 2019. However, the latter analysis was unadjusted for sampling design of the data due to small sample size. Again, the use of asset index as a measure of socio-economic status has its own limitation, as it may not necessarily show current socioeconomic status. The study did not adjust for the assumptions of confounding and population homogeneity when using the concentration index, therefore results maybe biased. However, the study is useful to serve as a guide for more detailed and elaborate future primary research.

## Conclusions

The study found socioeconomic status related inequalities in malaria prevalence to the disadvantage of poorer under-five children, highlighting the significant role played by socioeconomic status, maternal education, regional disparities, and rural residency. While Ghana has made progress in reducing the overall prevalence of malaria through the deployment of malaria vaccines and the distribution of insecticide-treated nets, there remains a crucial need for further action. It is essential to integrate these health initiatives with social policies that address the underlying socioeconomic inequalities in malaria prevalence. To bridge the inequality gap, it is imperative to tailor malaria control efforts to the needs of high-risk and underserved populations. This approach should harmonize epidemiological control measures with sustainable social and developmental policies.

## Supplementary Information

Additional file 1

## Data Availability

Data is available upon request at the Demographic and health survey website.https://dhsprogram.com/data/dataset_admin/login_main.cfm?CFID=562097&CFTOKEN=6937ffa0db78b551-7AA1FD16-9B2C-99D2-E5A2C5FAB09B71C8

## Abbreviations

CC: Concentration Curve
CDC: Centre for Disease Control and Prevention
CI: Concentration Index
CSDH: Commission of Social Determinants of Health Framework
DHS: Demographic and Health Survey
GMIS: Ghana Malaria Indicator Survey
GNMESP: Ghana National Malaria Elimination Strategic Plan
HREC: Human Research Ethics Committee
IPT: Intermittent Preventive Treatment
ITN: Insecticide-Treated Nets
LMICs: Lower Middle-Income Countries
NMEP: National Malaria Elimination Programme
PCA: Principal Component Analysis
RDT: Rapid Diagnostic Tests
SDGs: Sustainable Development Goals
SES: Socio-economic status
SSA: Sub-Saharan Africa
