# Supplementary material for: Socio-economic inequalities in malaria prevalence among under-five children in Ghana between 2016 and 2019: a decomposition analysis
Source: Malar J. 2025 May 8;24:147. doi: 10.1186/s12936-025-05349-y (PMC12063222; doi:10.1186/s12936-025-05349-y)
Supplement: Supplementary file 1 — Additional file 1 [file 12936_2025_5349_MOESM1_ESM.docx]

**Table A: Decomposition of Concentration Index for Malaria Prevalence for Under-Five Children in Ghana, 2019 (original analysis to the journal with proxy malaria outcome)**

| **Explanatory variables** | **Elasticities (SE)** | **Concentration indices (SE)** | **Contributions (SE)** | **Total contributions** | **Percentage of total**  **contribution** |
| --- | --- | --- | --- | --- | --- |
| **Wealth quintile** | | | | | |
| Poorest quintile (ref) | | | | | |
| Poorer quintile | -0.098 (0.058) * | -0.200 (0.023) *** | 0.019 (0.012) | **-0.133** | **59.38 %** |
| Middle quintile | -0.065 (0.045) | 0.217 (0.021) *** | -0.014 (0.009) |  |  |
| Richer quintile | -0.075 (0.041) * | 0.549 (0.017) *** | -0.041 (0.022) * |  |  |
| Richest quintile | -0.114 (0.041) *** | 0.852 (0.008) *** | -0.097 (0.035) *** |  |  |
| **Household net ownership** | 0.129 (0.194) | -0.014 (0.005) *** | -0.002 (0.003) | **-0.002** | **0.89%** |
| **Under-five net use** | -0.043 (0.126) | -0.094 (0.009) *** | 0.004 (0.012) | **0.004** | **- 1.78 %** |
| **National health insurance coverage for the child** | -0.084 (0.099) | 0.041 (0.011) *** | -0.004 (0.004) | **-0.004** | **1.78%** |
| **Residence** | | | | | |
| Rural(ref) | | | | | |
| Urban | -0.038 (0.113) | -0.243 (0.011) *** | 0.009 (0.028) | **0.009** | **-4.02%** |
| **Maternal Education** | | | | | |
| No formal education (ref) | | | | | |
| Primary | 0.018 (0.049) | -0.099 (0.028) *** | -0.002 (0.005) | **-0.016** | **7.14%** |
| Secondary | -0.093 (0.077) | 0.218 (0.018) *** | -0.020 (0.017) |  |  |
| Higher | 0.008 (0.015) | 0.739 (0.032) *** | 0.006 (0.011) |  |  |
| **Ethnicity** | | | | | |
| Akan (ref) | | | | | |
| Ga/Dangme | -0.002 (0.009) | 0.485 (0.057) *** | -0.001 (0.004) | **-0.01** | **4.46%** |
| Ewe | -0.018 (0.021) | 0.346 (0.033) *** | -0.006 (0.007) |  |  |
| Guan | -0.023 (0.009) ** | 0.078 (0.061) | -0.002 (0.002) |  |  |
| Mole Dagbani | 0.001 (0.133) | -0.219 (0.013) *** | -0.000 (0.029) |  |  |
| Grusi | -0.039 (0.017) ** | -0.185 (0.055) *** | 0.007 (0.004) * |  |  |
| Gurma | 0.034 (0.021) | -0.194 (0.055) *** | -0.007 (0.005) |  |  |
| Mande | -0.012 (0.004) *** | 0.066 (0.179) | -0.001 (0.002) |  |  |
| **Regions** | | | | | |
| Western (ref) | | | | | |
| Central | -0.013 (0.008) | 0.346 (0.034) *** | -0.005 (0.003) | **-0.053** | **23.66%** |
| Greater Accra | -0.041 (0.013) *** | 0.782 (0.017) *** | -0.032 (0.010) *** |  |  |
| Volta | -0.030 (0.025) | 0.171 (0.038) *** | -0.005 (0.004) |  |  |
| Eastern | -0.059 (0.023) ** | 0.393 (0.034) *** | -0.023 (0.009) ** |  |  |
| Ashanti | -0.095 (0.033) *** | 0.552 (0.026) *** | -0.053 (0.018) *** |  |  |
| Brong Ahafo | -0.077 (0.038) ** | 0.148 (0.033) *** | -0.011 (0.006) * |  |  |
| Northern | -0.189 (0.080) ** | -0.097 (0.026) *** | 0.018 (0.009) * |  |  |
| Upper East | - 0.008 (0.075) | -0.385 (0.032) *** | 0.003 (0.028) |  |  |
| Upper West | -0.185 (0.083) ** | -0.295 (0.028) *** | 0.055 (0.025) ** |  |  |
| **Age of Child** | | | | | |
| 0-12 months (ref) | | | | | |
| 13-24 months | 0.163 (0.040) *** | 0.003 (0.028) | 0.000 (0.004) | **0.004** | **-1.78%** |
| 25-36 months | 0.156 (0.039) *** | 0.037 (0.030) | 0.006 (0.005) |  |  |
| 36-48 months | 0.064 (0.034) * | -0.025 (0.031) | -0.001 (0.002) |  |  |
| 49-59 months | 0.051 (0.031) | -0.035 (0.033) | -0.001 (0.002) |  |  |
| **Age of Mother** | | | | | |
| 15-24 years (ref) | | | | | |
| 25-34 years | -0.048 (0.087) | 0.068 (0.015) *** | -0.003 (0.006) | **-0.006** | **2.68 %** |
| 35-44 years | -0.028 (0.055) | -0.033 (0.026) | 0.001 (0.002) |  |  |
| 45-49years | 0.019 (0.015) | -0.226 (0.091) ** | -0.004 (0.005) |  |  |
| **Residual** |  |  | -0.017 (0.014) | **-0.017** | **7.59 %** |
| **Explained contribution** |  |  |  | **-0.207***** | **92.41%** |
| **Total** |  |  |  | **-0.224** | **100%** |

*The estimated sample size was 1938; standard errors in parenthesis were bootstrapped using 1000 replications adjusting for sampling design.*

****, **, * indicate the statistical significance at 99%, 95%, and 90% confidence intervals.*

**Table B: Decomposition of Concentration Index for Malaria Prevalence for Under-Five Children in Ghana, 2019 (unadjusted analysis with malaria test)**

| **Explanatory variables** | **Elasticities (SE)** | **Concentration indices (SE)** | **Contributions (SE)** | **Total contributions** | **Percentage of total**  **Contribution (%)** |
| --- | --- | --- | --- | --- | --- |
| **Wealth quintile** | | | | | |
| Poorest quintile (ref) | | | | | |
| Poorer quintile | 0.033 (0.075) | -0.789 (0.025) *** | -0.026 (0.043) | **-0.015** | **32.61** |
| Middle quintile | 0.003 (0.032) | -0.383 (0.049) *** | -0.001 (0.014) |  |  |
| Richer quintile | 0.016 (0.019) | -0.000 (0.052) | -0.000 (0.001) |  |  |
| Richest quintile | 0.029 (0.016) * | 0.391 (0.048) *** | 0.012 (0.007) * |  |  |
| **Household net ownership** | -0.139 (0.138) | -0.016 (0.013) | 0.002 (0.003) | **0.002** | **-4.35** |
| **Under-five net use** | 0.112 (0.064) * | -0.108 (0.026) *** | -0.012 (0.007) | **-0.012** | **26.08** |
| **National health insurance coverage for the child** | -0.006 (0.038) | 0.043 (0.031) | -0.000 (0.002) | **-0.000** | **-** |
| **Residence** | | | | | |
| Rural (ref) | | | | | |
| Urban | 0.140 (0.069) ** | -0.266 (0.027) *** | -0.037 (0.018) ** | **-0.037** | **80.43** |
| **Maternal Education** | | | | | |
| No formal education (ref) | | | | | |
| Primary | 0.044 (0.021) ** | -0.087 (0.062) | -0.004 (0.004) | **-0.001** | **2.17** |
| Secondary | -0.001 (0.034) | 0.239 (0.039) *** | -0.000 (0.008) |  |  |
| Higher | 0.006 (0.009) | 0.768 (0.101) *** | 0.005 (0.007) |  |  |
| **Ethnicity** | | | | | |
| Akan (ref) | | | | | |
| Ga/Dangme | -0.003 (0.004) | 0.654 (0.108) *** | -0.002 (0.003) | **-0.011** | **23.91** |
| Ewe | -0.000 (0.008) | 0.246 (0.079) *** | -0.000 (0.002) |  |  |
| Guan | 0.000 (0.001) | 0.395 (0.055) *** | 0.000 (0.000) |  |  |
| Mole Dagbani | 0.035 (0.089) | -0.319 (0.039) *** | -0.011 (0.029) |  |  |
| Grusi | -0.015 (0.015) | -0.125 (0.168) | 0.002 (0.005) |  |  |
| Gurma | -0.004 (0.014) | -0.451 (0.068) *** | 0.002 (0.007) |  |  |
| Mande | -0.004 (0.003) | 0.566 (0.051) *** | -0.002 (0.002) |  |  |
| **Regions** | | | | | |
| Western (ref) | | | | | |
| Central | 0.005 (0.002) ** | 0.347 (0.093) *** | 0.002 (0.001) | **0.012** | **-26.08** |
| Greater Accra | 0.002 (0.003) | 0.831 (0.041) *** | 0.002 (0.002) |  |  |
| Volta | -0.004 (0.009) | 0.063 (0.086) | -0.000 (0.001) |  |  |
| Eastern | 0.001 (0.007) | 0.305 (0.125) ** | 0.000 (0.002) |  |  |
| Ashanti | -0.017 (0.014) | 0.553 (0.082) *** | -0.009 (0.007) |  |  |
| Brong Ahafo | 0.001 (0.023) | 0.079 (0.111) | 0.000 (0.002) |  |  |
| Northern | 0.018 (0.026) | -0.279 (0.085) *** | -0.005 (0.007) |  |  |
| Upper East | -0.029 (0.048) | -0.375 (0.059) *** | 0.011 (0.018) |  |  |
| Upper West | -0.020 (0.034) | -0.530 (0.065) *** | 0.011 (0.018) |  |  |
| **Age of Child** | | | | | |
| 0-12 months (ref) | | | | | |
| 13-24 months | 0.029 (0.029) | 0.154 (0.059) *** | 0.004 (0.005) | **-0.002** | **4.34** |
| 25-36 months | 0.038 (0.031) | -0.043 (0.056) | -0.002 (0.003) |  |  |
| 36-48 months | 0.034 (0.019) | -0.061 (0.091) | -0.002 (0.004) |  |  |
| 49-59 months | 0.018 (0.016) | -0.096 (0.092) | -0.002 (0.003) |  |  |
| **Age of Mother** | | | | | |
| 15-24 years (ref) | | | | | |
| 25-34 years | 0.023 (0.042) | 0.089 (0.041) ** | 0.002(0.004) | **-0.008** | **17.39** |
| 35-44 years | 0.026(0.028) | -0.141 (0.061) ** | -0.004(0.005) |  |  |
| 45-49years | 0.012 (0.008) | -0.452 (0.158) *** | -0.006 (0.005) |  |  |
| **Explained contribution** |  |  | **-0.072****(0.031) | **-0.072**** | **156.52** |
| **Residual** |  |  | **0.026** (0.018) | **0.026** | **-56.52** |
| **Total** |  |  |  | **-0.046**** | **100** |

*The estimated sample size was 256; standard errors in parenthesis were bootstrapped using 1000 replications adjusting for sampling design.*

****, **, * indicate the statistical significance at 99%, 95%, and 90% confidence intervals.*

*Note: One or more parameters could not be estimated in 599 bootstrap replicates; standard-error estimates include only complete replications.*

**Table C: Decomposition of Concentration Index for Malaria Prevalence for Under-Five Children in Ghana, 2016 (uadjusted data with just malaria test results)**

| **Explanatory variables** | **Elasticities (SE)** | **Concentration indices (SE)** | **Contributions (SE)** | **Total contributions** | **Percentage of total**  **contribution** |
| --- | --- | --- | --- | --- | --- |
| **Wealth quintile** | | | | | |
| Poorest quintile (ref) | | | | | |
| Poorer quintile | 0.112 (0.058) * | -0.797(0.025) *** | -0.089 (0.045) * | **-0.089** |  |
| Middle quintile | 0.042 (0.039) | -0.392 (0.051) *** | -0.016 (0.015) |  |  |
| Richer quintile | 0.018 (0.024) | 0.007 (0.056) | 0.000 (0.001) |  |  |
| Richest quintile | 0.039 (0.019) ** | 0.396 (0.050) *** | 0.016 (0.007) ** |  |  |
| **Household net ownership** | -0.119 (0.115) | -0.021 (0.010) ** | 0.003 (0.002) | **0.003** |  |
| **Under-five net use** | -0.008 (0.062) | -0.107 (0.025) *** | 0.001 (0.006) | **0.001** |  |
| **National health insurance coverage for the child** | 0.039 (0.052) | 0.040 (0.023) * | 0.002 (0.002) | **0.002** |  |
| **Residence** | | | | | |
| Rural (ref) | | | | | |
| Urban | 0.014 (0.060) | -0.255 (0.027) *** | -0.003 (0.015) | **-0.003** |  |
| **Maternal Education** | | | | | |
| No formal education (ref) | | | | | |
| Primary | 0.011(0.024) | -0.142 (0.058) ** | -0.002 (0.003) | **0.009** |  |
| Secondary | 0.033 (0.030) | 0.224 (0.042) *** | 0.007 (0.006) |  |  |
| Higher | 0.005 (0.013) | 0.731 (0.064) *** | 0.004 (0.009) |  |  |
| **Ethnicity** | | | | | |
| Akan (ref) | | | | | |
| Ga/Dangme | 0.001 (0.004) | 0.377 (0.209) * | 0.001 (0.001) | **0.004** |  |
| Ewe | 0.003(0.009) | 0.204 (0.079) ** | 0.001 (0.001) |  |  |
| Guan | 0.008 (0.006) | 0.141 (0.231) | 0.001 (0.002) |  |  |
| Mole Dagbani | -0.004 (0.061) | -0.397 (0.050) *** | 0.002 (0.024) |  |  |
| Grusi | 0.006 (0.020) | -0.277 (0.137) ** | -0.002 (0.006) |  |  |
| Gurma | -0.000 (0.016) | -0.263 (0.109) ** | 0.000 (0.005) |  |  |
| Mande | -0.003 (0.011) | -0.462 (0.144) *** | 0.001 (0.001) |  |  |
| **Regions** | | | | | |
| Western (ref) | | | | | |
| Central | 0.001(0.003) | 0.156 (0.077) ** | 0.000 (0.001) | **0.048** |  |
| Greater Accra | -0.003 (0.005) | 0.736 (0.057) *** | -0.002 (0.003) |  |  |
| Volta | -0.013 (0.010) | 0.270 (0.089) *** | -0.003 (0.002) |  |  |
| Eastern | -0.000 (0.011) | 0.189 (0.088) ** | -0.000 (0.002) |  |  |
| Ashanti | -0.014 (0.011) | 0.535 (0.094) *** | -0.007 (0.006) |  |  |
| Brong Ahafo | -0.026 (0.022) | 0.102 (0.080) | -0.003 (0.003) |  |  |
| Northern | -0.018 (0.026) | -0.352 (0.094) *** | 0.006 (0.009) |  |  |
| Upper East | -0.068 (0.061) | -0.563 (0.060) *** | 0.039 (0.035) |  |  |
| Upper West | -0.053 (0.036) | -0.330 (.083) *** | 0.018 (0.012) |  |  |
| **Age of Child** | | | | | |
| 0-12 months (ref) | | | | | |
| 13-24 months | 0.060 (0.023) ** | 0.012 (0.068) | 0.001 (0.004) | **-0.001** |  |
| 25-36 months | 0.086 (0.031) *** | -0.018 (0.063) | -0.002 (0.005) |  |  |
| 36-48 months | 0.052 (0.026) * | -0.062 (0.075) | -0.003 (0.004) |  |  |
| 49-59 months | 0.071 (0.023) *** | 0.044 (0.081) | 0.003 (0.005) |  |  |
| **Age of Mother** | | | | | |
| 15-24 years (ref) | | | | | |
| 25-34 years | -0.019 (0.046) | -0.043 (0.033) | 0.001 (0.002) | **0.004** |  |
| 35-44 years | -0.003 (0.024) | 0.082 (0.058) | -0.000 (0.002) |  |  |
| 45-49years | -0.006 (0.008) | -0.437 (0.171) ** | 0.003 (0.004) |  |  |
| **Explained Contribution** |  |  | -0.024 (0.032) | **-0.024** |  |
| **Residual** |  |  | 0.015 (0.019) | **0.015** |  |
| **Total** |  |  |  | **-0.009** |  |

*The estimated sample size was 252; standard errors in parenthesis were bootstrapped using 1000 replications adjusting for sampling design.*

****, **, * indicate the statistical significance at 99%, 95%, and 90% confidence intervals.*

*Note: One or more parameters could not be estimated in 2 bootstrap replicates; standard-error estimates include only complete replications.*
